# Supplementary material for: Exploring Nurses’ Quit Intentions: A Structural Equation Modelling and Mediation Analysis Based on the JD-R and Social Exchange Theories
Source: Healthcare (Basel). 2025 Mar 21;13(7):692. doi: 10.3390/healthcare13070692 (PMC11988909; doi:10.3390/healthcare13070692)
Supplement: Supplementary file 1 [file healthcare-13-00692-s001.zip › healthcare-3374980-supplementary.pdf]

Supplementary Material

Figure S1. Full Path analysis model with unstandardized coefficients and SE.

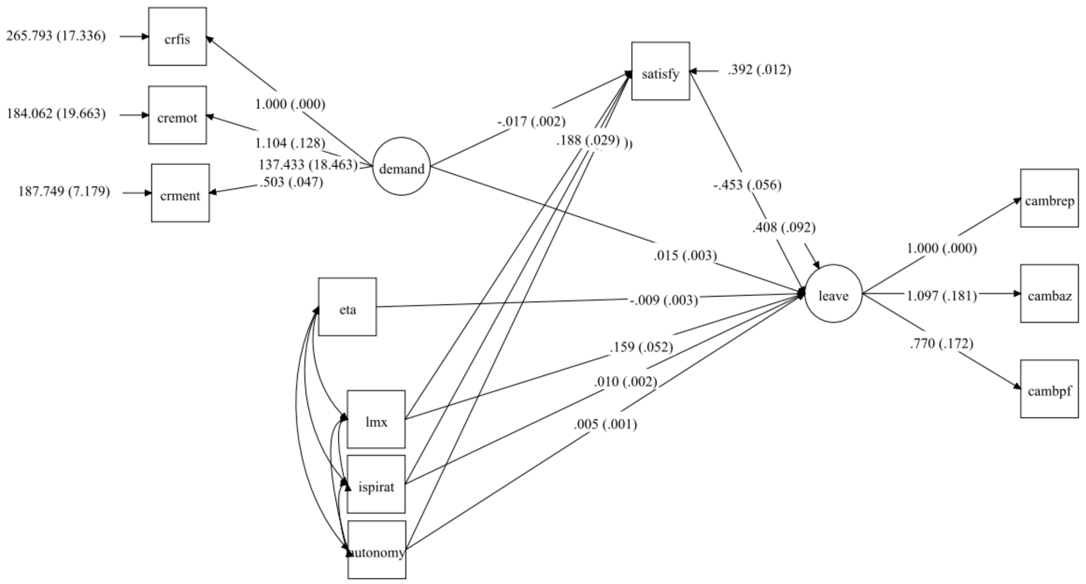

Table S1. Complete model Confidence Interval information.

Variable	Lower .5%	Lower 2.5%	Lower 5%	Estimate	Upper 5%	Upper 2.5%	Upper .5%
**LEAVE ON**							
DEMAND	0,007	0,009	0,01	0,015	0,02	0,021	0,023
ISPIRAT	0,005	0,006	0,007	0,01	0,013	0,014	0,015
LMX	0,025	0,057	0,074	0,159	0,245	0,261	0,293
AUTONOMY	0,001	0,002	0,003	0,005	0,007	0,007	0,008
SATISFY	-0,597	-0,563	-0,545	-0,453	-0,361	-0,343	-0,309
AGE	-0,016	-0,014	-0,013	-0,009	-0,004	-0,004	-0,002
**SATISFY ON**							
DEMAND	-0,021	-0,02	-0,02	-0,017	-0,014	-0,014	-0,012
ISPIRAT	-0,012	-0,012	-0,011	-0,01	-0,008	-0,008	-0,008
LMX	0,113	0,131	0,14	0,188	0,235	0,244	0,262
AUTONOMY	-0,008	-0,007	-0,007	-0,006	-0,005	-0,004	-0,004
